# Distance‐Dependent Energy Transfer Between Organic Fluorophores and Single‐Walled Carbon Nanotubes

**DOI:** 10.1002/anie.202520411

**Published:** 2026-02-08

**Authors:** Izabela Kamińska, Justus T. Metternich, Alan M. Szalai, Carolin Smidoda, Sayantani Chakraborty, Lela Vukovic, Sebastian Kruss, Philip Tinnefeld

**Affiliations:** ^1^ Department of Chemistry and Center for NanoScience Ludwig Maximilian University of Munich Munich Germany; ^2^ Institute of Physical Chemistry of the Polish Academy of Sciences Warsaw Poland; ^3^ Department of Chemistry and Biochemistry Ruhr‐University Bochum Bochum Germany; ^4^ Biomedical Nanosensors Fraunhofer Institute for Microelectronic Circuits and Systems Duisburg Germany; ^5^ Centro de Investigaciones en Bionanociencias (CIBION) Consejo Nacional de Investigaciones Científicas y Técnicas (CONICET) Ciudad Autónoma de Buenos Aires Argentina; ^6^ Department of Chemistry and Biochemistry University of Texas El Paso El Paso Texas USA; ^7^ Computational Science Program University of Texas El Paso El Paso Texas USA; ^8^ Bioinformatics Program University of Texas El Paso El Paso Texas USA

**Keywords:** dsDNA, energy transfer, fluorescence quenching, single molecules, single‐walled carbon nanotubes

## Abstract

Single‐walled carbon nanotubes (SWCNTs) are promising optical biosensing platforms due to their intrinsic near‐infrared fluorescence and environmental sensitivity. While DNA‐SWCNT hybrids have been widely studied, the structural arrangement of double‐stranded DNA (dsDNA) on SWCNTs and its impact on exciton–fluorophore interactions remain insufficiently characterized. Here, we introduce carbon nanotube energy transfer with vertical nucleic acids (CNETvNA), in which fluorophores are positioned at defined distances from SWCNTs using guanine‐defect anchored capture sequences hybridized with complementary oligonucleotides. By systematically varying the duplex length from 12 to 24 base pairs, we probe the distance dependence of dye–SWCNT interactions at the single‐molecule level. Fluorescence lifetime imaging microscopy reveals efficient quenching of ATTO542 and ATTO643 dyes, with lifetime distributions reflecting heterogeneous duplex conformations. Molecular dynamics simulations demonstrate that dsDNA duplexes adopt a predominantly perpendicular orientation relative to the SWCNT axis, with increasing tilt and conformational variability at longer lengths. Combining experimental and computational results, we establish a distance dependence of d^−^^5^ with 7.4 ± 0.7 nm for 50% quenching efficiency, consistent with theoretical predictions for point dipole donors and 1D acceptors. These findings provide structural insights into DNA‐SWCNT conjugates and establish CNETvNA as a rational design principle for SWCNT‐based biosensors.

## Graphical abstract

We demonstrate that the dsDNA part adopts a perpendicular orientation to the SWCNT and present a distance‐dependent energy transfer of single fluorescent dyes to SWCNTs. Carbon nanotube energy transfer (CNET) obeys a d^−5^ distance dependence with a characteristic distance of 7.4 nm (± 0.7 nm) for 50 % energy transfer.

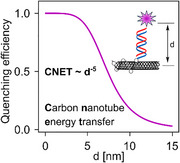

## 1 Introduction

Low‐dimensional materials are promising for a wide range of applications, and energy transfer processes have attracted significant attention in fundamental science and technological applications. Among these materials, single‐walled carbon nanotubes (SWCNTs) are known for their rich optoelectronic properties. Semiconducting SWCNTs are 1D nanomaterials that are photostable and fluoresce in the near‐infrared (NIR) tissue transparency region [1]. Their optical properties are governed by excitons, electron‐hole pairs with a dissociation energy of up to 500 meV, that are created upon excitation. [2, 3] In (6,5)‐SWCNTs, the size of excitons is estimated between 2 and 13 nm. During a lifetime of 10–70 ps, [4] these quasi‐particles diffuse approx. 90 nm. [5] Interactions with their local environment render the fluorescence of SWCNTs sensitive to their chemical surroundings [6, 7] and form the basis for applications in (bio)sensing and (bio)photonics. [8, 9, 10, 11, 12, 13, 14, 15]

To create sensors, SWCNTs have been functionalized with different polymers that form complex interfaces [16, 17], decorated with defects (color centers) [18], and rationally assembled by tethering recognition units to the surface of the SWCNT. [19] Most often, single‐stranded DNA (ssDNA) is used to disperse SWCNTs [20, 21, 22, 23, 24]. The interaction between DNA and SWCNTs is governed by π–π interactions between the DNA bases and the π‐system of the SWCNT, thereby exposing the phosphate backbone of the DNA. Due to the nature of these interactions, the interface of DNA‐SWCNT hybrids is highly flexible, and interactions in crowded environments can lead to strong reorganization of the SWCNT corona [25]. For stable covalent modification, so‐called guanine quantum defects (*G* ^d^) can be introduced [26, 27, 28]. When only parts of the sequence contain guanine, only these positions are capable of forming a covalent bond. Connected capture sequences remain flexible and allow for hybridization‐based assembly and a rational bottom‐up design that provides an efficient way for functionalization [29]. SWCNTs are highly efficient quenchers of organic dyes through exciton–fluorophore interactions, and this property has been widely exploited in designing fluorescent probes and biosensors [30, 31, 32, 33]. Conversely, fluorophores can also modulate the intrinsic near‐infrared fluorescence of SWCNTs [26, 34, 35, 36]. Such energy transfer and quenching effects provide a versatile mechanism to tailor the optical response of both dye‐ and SWCNT‐based sensing schemes. For example, a better understanding could inform the design of sensors that switch on/off when an analyte interacts with dye‐SWCNT hybrids [37].

While hybrid materials composed of ssDNA and SWCNTs been extensively studied, [20, 21, 38] comparatively little is known about the precise orientation of double‐stranded DNA (dsDNA) duplexes linked to the SWCNT [39]. Does the double‐stranded region adopt a vertical orientation on SWCNTs as was recently found for graphene [40]? Besides answering this question, the distance dependence of the energy transfer from organic fluorophores to SWNCTs has not been characterized. Energy transfer between two dye molecules is described by the Förster theory and follows the famous r^−6^ scaling law. This distance dependence must be adapted for different dimensionalities of the acceptor. Energy transfer to a 2D material, such as graphene, or to a sheet of dyes or nanoparticles, obeys r^−4^ distance dependence [40, 41, 42]. Other scaling laws are r^−3^ distance dependence for energy transfer to a volumetric metal [43] or the predicted r^−5^ distance dependence for energy transfer to a line of transition dipoles, such as a wire [44, 45]. Both the orientation of dsDNA and the distance dependence of energy transfer are crucial for understanding and optimizing the performance of SWCNT‐based sensors, as they directly influence the efficiency of signal transduction and the specificity of molecular recognition. Additionally, it is necessary to understand the design and architecture of hybrid materials based on DNA and SWCNTs.

## 2 Results and Discussion

Here, we present SWCNT energy transfer (CNET) with vertical nucleic acids (CNETvNA). Using hybridization between labeled oligos and capture strands that are tethered to SWCNTs using anchor sequence *G* ^d^, we reveal the distance dependence of CNET. The experimental results are supplemented with a set of molecular dynamics (MD) simulations that elucidate the orientation of the duplex on the SWCNT and provide the foundation for the distance dependence of the energy transfer phenomenon.

For specific labeling of SWCNTs with dsDNA, we modified the SWCNT surface with a DNA that features a guanine‐containing anchor sequence (GT)_15_ and a guanine‐free capture sequence [28, 29, 46, 47]. To increase the homogeneity of the DNA‐SWCNT interaction, the anchor sequence was covalently linked to the SWCNT by introducing guanine defects by an established procedure using rose Bengal (20 µM) and green light [29].

**FIGURE 1:**
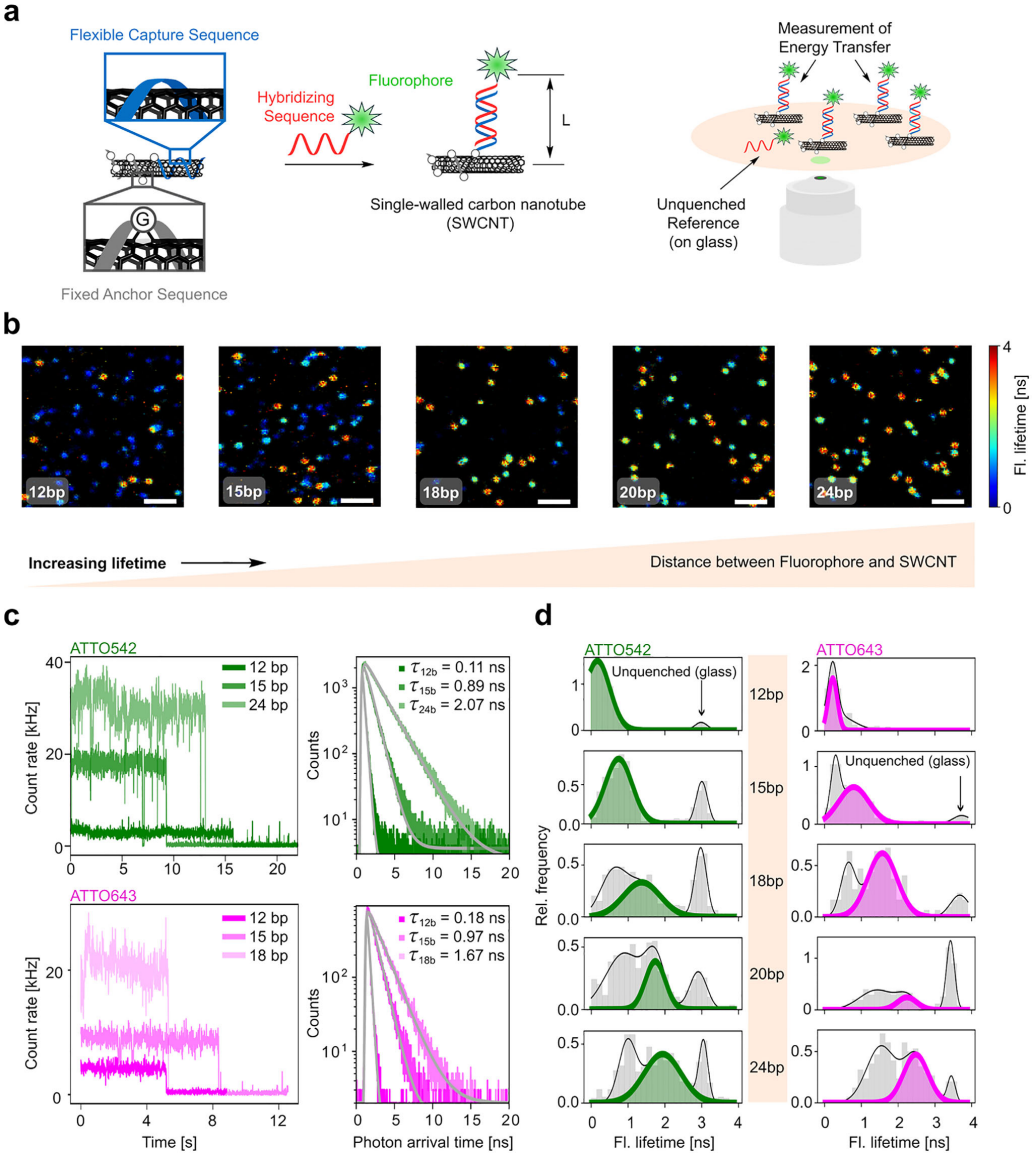
Distance‐dependent energy transfer between fluorophores and single‐walled carbon nanotubes (SWCNT). a) Schematic illustration of a SWCNT with a capture (S0) (blue) and an anchor (S1) sequence (gray) and the imaging setup. Quantum defects were introduced to fixate the anchors. The capture sequence was truncated to different lengths (L). Hybridization of a labelled oligonucleotide (red) leads to a defined positioning of the fluorophore (green, ATTO542). b) Fluorescence lifetime images of a dye molecule ATTO542 hybridized on capture sequences with different lengths. Scale bars are 2 µm. c) Exemplary single‐molecule fluorescence time traces for ATTO542 and ATTO643 dyes bound to DNA duplexes of variable lengths (left), along with their corresponding fluorescence decay curves (right). Monoexponential fits of the decay curves are shown in gray solid lines. d) Fluorescence lifetime histograms for traces acquired with ATTO542 (green) or ATTO643 (magenta) attached to a 5’ end of an oligonucleotide that is hybridized on SWCNTs with (from top to bottom) 12b, 15b, 18b, 20b, and 24b long capture sequences.

To position organic fluorophores at different distances, the capture sequence length (S0) was varied from 12b to 15b, 18b, 20b, and 24b (Figure 1a, Table S1). Upon DNA hybridization, the nucleotides are no longer available for stacking interactions with the SWCNTs, and the resulting dsDNA should therefore protrude from the SWCNT. For single‐molecule inquiry of energy transfer in dye–SWCNT pairs, suspensions of SWCNTs functionalized with different capture sequences were incubated with the complementary oligonucleotides S1 labeled with either ATTO542 or ATTO643 (Figure 1a, Table S2). We used a concentration of complementary strands that led to an average of a single dye per SWCNT, and only used measurements where the fluorescence signal exhibited a single photobleaching step. The resulting duplexes were immobilized on cleaned glass coverslips for imaging. While the capture sequence length was systematically varied from 12b to 24b, the fluorophore‐labeled imager sequence was always a 24b ssDNA. When the capturing strand was shorter than 24b, an overhang was left close to the SWCNTs, which was also adsorbed to the nanotube surface. For quantifying potential energy transfer, we measured the fluorescence lifetime of ATTO542 and ATTO643 dyes using 532 and 636 nm laser excitations, respectively (see also Supporting Information for sample preparation, experimental conditions, and data analysis).

In Figure 1b, we show single‐molecule fluorescence lifetime imaging microscopy (FLIM) maps for the different systems labeled with a dye ATTO542. The FLIM maps show, on average, lower fluorescence lifetimes (i.e., more efficient quenching) for shorter capture sequences, confirming that the dye is placed closer to the SWCNT in those cases.

To quantitatively analyze this effect, we recorded fluorescence lifetime decay curves for hundreds of single molecules by gathering all detected photons before photobleaching. In Figure 1c, representative single‐molecule time traces along with their fluorescence decays and monoexponential fits are shown for the different systems. The distributions of fluorescence lifetimes for molecules corresponding to each DNA duplex length and dye are shown in Figure 1d. Remarkably, fluorescence lifetime values ranging from 0.1 to 3–3.5 ns were obtained, confirming that the designed probes are adequate to study a broad range of energy transfer efficiencies from these fluorophores to SWCNTs. In nearly all cases, a peak corresponding to unquenched dyes is evident (fluorescence lifetimes close to 3 ns for ATTO542 and 3.5 ns for ATTO643), which can be explained by DNA that was non‐specifically bound to glass instead of to SWCNTs. Beyond these peaks, both dyes exhibit a clear shift toward longer fluorescence lifetimes as the duplex length increases, consistent with increasing dye–SWCNT distances. For each dsDNA length, the observed distribution is typically broader than that of unquenched dyes. This is mainly related to static heterogeneity, as not all DNA adopts the same conformation on the SWCNTs, whereas little fluorescence lifetime fluctuations are observed within the time traces (Figure 1c).

To assess whether a perpendicular orientation of dsDNA occurs here, as we previously observed for graphene, we focused on the less‐quenched subpopulations in each system, since such an orientation would position the dye as far as possible from the SWCNTs. To achieve this, we performed multi‐peak Gaussian fits on the histograms from Figure 1d. The peaks at ∼3 ns for ATTO542 and ∼3.5 ns for ATTO643 were attributed to unquenched dyes (see Figure S12 and S13 for the reference measurements). Among the remaining peaks, those corresponding to higher fluorescence lifetimes were assigned to the (potentially) perpendicular configuration (colored in green and magenta for ATTO542 and ATTO643, respectively). Subpopulations with shorter lifetimes correspond to cases where the duplex showed higher tilting, therefore presenting, on average, shorter distances. Another possible explanation for a shorter fluorescence lifetime would be a partial unzipping of the duplex at the interface with the SWCNT, something already observed for graphene [48, 49, 50]. To determine the appropriate number of Gaussian functions for fitting, we applied constraints on the width of a single population and selected the minimal number of functions that provided a well‐fitted model, as assessed by the Akaike Information Criterion (AIC) (see Methods for details). Notably, fluorophores positioned near the distance corresponding to 50% quenching efficiency (i.e., lifetimes around 1.5 and 1.8 ns for ATTO542 and ATTO643) inherently exhibit a broader distribution of lifetimes. This is due to the high sensitivity of quenching in this range, where small height fluctuations lead to significant variations in quenching efficiency and, consequently, in fluorescence lifetime. To account for this effect, we allowed a relatively broad range for the width of a single population (0.05 to 0.5 ns). Finally, we converted fluorescence lifetime into quenching efficiency using the formula $\eta =1-\frac{\tau }{\tau_{0}}$ where τ is the actual fluorescence lifetime and τ_0_ is the lifetime in the absence of fluorescence quenching, which was obtained from the same dataset (see Table S3).

**FIGURE 2:**
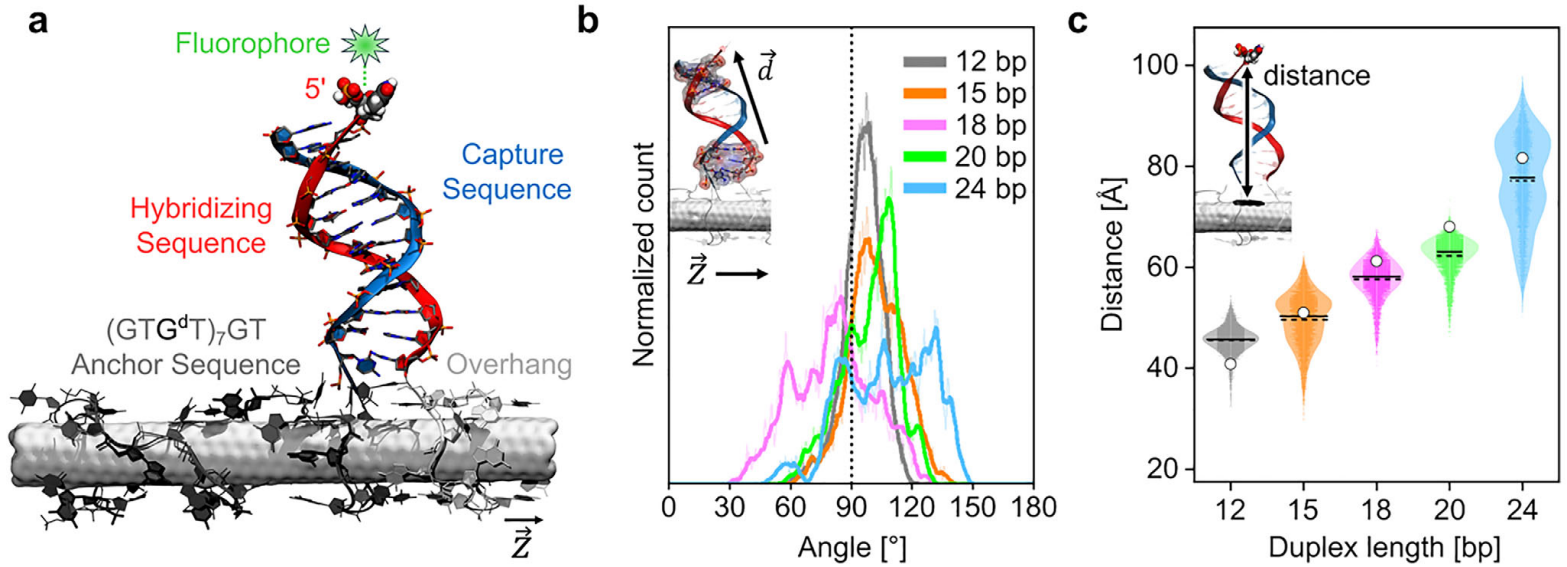
Duplex tip distances and orientations in molecular dynamics simulations. a) Structure of the S1‐12 bp system after equilibration in MD simulations. The SWCNT is shown as a white surface. The anchor sequence and the overhang segment of the complementary strand are shown in dark gray and light gray, respectively, using licorice and ribbon representations. The duplex is shown in blue (capture sequence) and red (complementary sequence) using licorice and cartoon representations. The 5′‐end nucleotide of the complementary strand, which is not base‐paired, is shown with van der Waals spheres. Atoms are colored as: C (gray), N (blue), O (red), P (orange), H (white). b) Angles between duplexes and the SWCNT axis over 400 ns of MD simulations. The dotted line marks the orthogonal orientation (90°). (inset) A schematic showing two vectors used to calculate angles between duplexes and the SWCNT axis using the dot product relationship. c) Distances between the 5′‐end nucleotides of the complementary sequences and the nearest points on the SWCNT surface, over 400 ns of MD simulations. Empty circles mark the estimated duplex lengths, based on a 0.34 nm rise per base pair. Solid and dashed horizontal lines in violin plots indicate the median and mean values of distributions, respectively. (Inset) A schematic defining the distance measured in each system.

While energy transfer efficiencies can be extracted from single‐molecule fluorescence lifetime acquisitions, we cannot directly translate these values into distances, as neither the exact orientation of the dsDNA with respect to the SWCNT is known nor the exact scaling law of the energy transfer from the dye to the SWCNT. To examine the structure and dynamics of DNA‐SWCNT conjugates at atomic resolution, we performed all‐atom MD simulations of five systems that varied in duplex length. Each system included a DNA anchor covalently bound to a (6,5)‐SWCNT via guanine defect chemistry, using the sequence (GTG^d^T)_7_GT. This anchor was extended by a single‐stranded DNA capture sequence, which hybridized with a separate 24‐nucleotide complementary strand (S1) to form duplexes of 12, 15, 18, 20, or 24 base pairs (bp). In all systems except the one with a 24 bp duplex, the 3′‐end of the complementary strand formed an unpaired overhang that wrapped around the SWCNT surface. A representative structure of the equilibrated 12 bp system is shown in Figure 2a. After 400 ns of simulation, the 12 bp duplex adopted a nearly orthogonal orientation relative to the SWCNT axis, with a slight tilt towards the anchor strand. Most of the other systems showed more pronounced tilting of the duplexes, with the 18 and 20 bp duplexes tilting slightly toward the overhang sequence, and the 15 and 24 bp duplexes tilted more significantly toward the anchor strand (Figure S14).

To quantify the duplex orientations, we calculated the angle between the duplex axis and the long axis of the SWCNT over the whole MD trajectories. As shown in Figure 2b, the 12 bp duplex maintained a narrow angular distribution centered around 90° to 100°, reflecting its near‐orthogonal orientation with a slight tilt toward the anchor strand. The 15 bp and 20 bp duplexes had broader angular distributions, still favoring tilting toward the anchor. The 18 bp and the 24 bp duplexes exhibited the widest‐angle distribution, ranging from 58° to 114° and 85° to 135°, respectively, suggesting increased flexibility. This greater angular variability in the 24 bp duplex likely arises from the absence of a 3′ overhang, which reduces steric constraints and allows more conformational freedom. In contrast, shorter duplexes with flanking overhangs and anchors are structurally restricted by DNA wrapping on both sides of the duplex (see also Figure S16 and supplementary discussion in SI).

Because the dye is attached to the 5′‐end of the complementary strand in experiments, the distance between this 5′‐end and the SWCNT surface is a critical structural parameter. We calculated these distances over the simulation trajectories (Figure 2c). On average, the distance increased with duplex length, consistent with the expectations based on a 0.34 nm rise per base pair. However, the distance distributions varied in width across systems. The 12 bp duplex showed the narrowest distribution, in agreement with the experimental results shown for fluorescence lifetimes (Figure 1d), while the 24 bp duplex had a broad, bimodal distribution, reflecting greater conformational variability. Some differences in the widths of the distance distributions can also be attributed to the stochasticity of the conformational sampling in MD simulations.

The average distance of the 5′‐end from the SWCNT in the 12 bp system was 4.5 nm, which is greater than the estimated duplex length of 4.08 nm. This deviation likely arises from the duplex resting on another 1–2 DNA nucleotides at the SWCNT surface, and from partial unpairing of the 5′‐end nucleotide at the duplex terminal, which was unbound for 88% of the simulation time (Table S6). For the 18 bp duplex system, the average distance of the 5’‐end from the SWCNT was comparable to the estimated duplex length (both ∼5.7 nm). In contrast, the other systems had slightly shorter average/median distances between the 5′‐end of the duplex and the SWCNT surface than expected, which can be due to the increased duplex tilting toward the SWCNT surface. This interpretation is supported by the correlation between the duplex tilt angle and the 5′‐end to SWCNT distance shown in Figure S15.

The predominantly perpendicular orientation of the dsDNA observed in our MD simulations then allowed us to correlate the fluorescence lifetimes of single dyes attached to one of the terminal bases of DNA duplexes (12, 15, 18, 20, and 24 bp) with their actual distances to the SWCNT surface. This, in turn, enables us to determine, for the first time, the distance‐dependent quenching parameters for dyes positioned near the SWCNTs, using the equation for quenching efficiency $\eta =\frac{1}{1+{\left(\frac{d}{d_{0}}\right)}^{5}}$, where d is the distance between a dye and SWCNT, and *d*_0_ is the distance of the 50% energy transfer efficiency to SWCNT.

**FIGURE 3:**
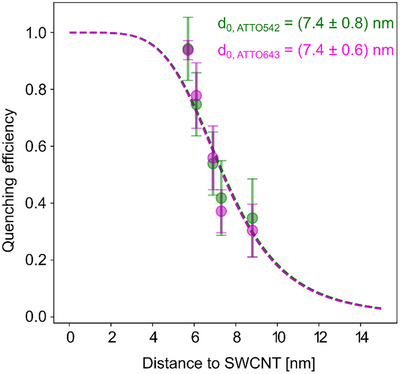
Distance dependent of energy transfer between organic fluorophores and SWCNTs (carbon nanotube energy transfer: CNET). The dashed lines are fits to the d^−5^ power law.

Figure 3 presents the distance dependence of quenching efficiency for the two studied dyes. The mean quenching efficiencies were derived from the peak positions of the less‐quenched populations in Figure 1d, with error bars obtained from the corresponding standard deviations. The distance to the SWCNT was estimated by combining the mean values obtained from MD simulations for each duplex (4.6, 5.0, 5.8, 6.2, and 7.7 nm) with an additional ∼1.1 nm contribution from the linker and dye [40]. Given that both dyes are negatively charged, the six‐carbon linker and dye are expected to extend linearly from the duplex, repelled by the negatively charged dsDNA backbone. Remarkably, both curves follow a d^−5^ power law (the sum of squared residuals for this model was 0.29, against 0.51 for d^−4^ and 0.31 for d^−6^), with almost identical characteristic distances of 7.4 nm. Using the formalism of Förster resonance energy transfer (FRET), it is possible to estimate the ratio between the Förster distance or radius (R0) of these two dyes by computing the spectral overlap (J) between each of them and the SWCNTs and by also considering their fluorescence quantum yields (QY): R_0(ATTO542)_/R_0(ATTO643)_ = (J_ATTO542_·QY_ATTO542_)/(J_ATTO643_·QY_ATTO643_)^(⅙). This expression assumes an identical refractive index and freely rotating fluorophores so that the orientation factor cancels. In Figure S4, we show the normalized emission spectra of the two fluorophores overlayed with the absorption spectrum of (G^d^T)^15^‐S0‐12b, all of which were used to calculate J. Notably, employing the QY values provided by the supplier ATTO‐tec, the obtained ratio R_0(ATTO542)_/R_0(ATTO643)_ is 0.99, meaning that the Förster radius is expected to be almost identical. While this was confirmed in our experiments, other dyes with different quantum yields or spectral properties may yield R0 values that deviate from those reported here. On the other hand, the d^−5^ dependence is well‐expected for the energy transfer from a point‐like donor and a 1D acceptor [44, 45]. Hernández‐Martínez and Govorov [44] have formally shown that the energy transfer from point‐like donors to nanowires follows a d^−5^ dependence when the distance between them is larger than the wire's diameter, as it occurs in our experiments. Additionally, they demonstrate that the efficiency of the energy transfer depends on orientational factors. In the case of carbon nanotubes, only donor dipoles with orientations that strongly couple to the longitudinal axis of the tubes will contribute to the energy transfer process. As expressed above, since our dyes are freely rotating, the measured distance dependence is the result of the averaging of all possible conformations. The d^−5^ power law contrasts with the dependencies found for 3D (d^−3^), [43] 2D (d^−4^), [41, 42, 51] and punctual (molecular, e.g., FRET; d^−6^) acceptors [52, 53, 54].

## 3 Conclusion

In summary, we present distance‐dependent energy transfer of single fluorescent dyes to SWCNTs. A precise arrangement of dyes was achieved by using a double‐stranded to single‐stranded DNA junction in which single‐stranded DNA acts as an anchor that wraps the SWCNTs and which is consolidated by (covalent) guanine (quantum defects) bonds. Using molecular dynamics simulations, we demonstrate that the dsDNA part adopts a mostly perpendicular orientation. In line with this, fluorescence lifetime analysis of single‐molecule signals reveals a distribution of energy transfer efficiencies with a profound population representing the perpendicular orientation, which provides a valuable insight for the assembly of higher‐dimensional materials based on SWCNTs and nucleic acids. The energy transfer process obeys a d^−5^ distance dependence with a characteristic distance of 7.4 nm (± 0.7 nm) for 50% energy transfer. The similar distance dependence for the green and red emitting dyes is surprising, as the spectral overlap of donor emission with the SWCNT‐acceptor absorption should be different for the different dyes. The results indicate that conformational changes in the dynamic range < 10 nm of the range (Figure 3) could be used for molecular sensing, for example, using aptamers or antibodies that are on the same order of magnitude in size. Overall, our study provides both a new level of structural control for arranging objects relative to SWCNTs using perpendicular dsDNA linkers as well as important insights regarding energy transfer between fluorophores and SWCNTs that can be exploited for biomolecular sensing applications. Among others, we anticipate that these structural and photophysical insights inform future rational design of the SWCNT corona, DNA‐based bioconjugation techniques, as well as the creation of powerful turn‐on/turn‐off sensors.

## Conflict of Interests

The authors declare no conflict of interest.

## Supporting information




**Supporting File 1**: anie71352‐sup‐0001‐SuppMat.docx.

## Data Availability

The data that support the findings of this study are available from the corresponding author upon reasonable request.
