# Supplementary material for: Distance‐Dependent Energy Transfer Between Organic Fluorophores and Single‐Walled Carbon Nanotubes
Source: Angew Chem Int Ed Engl. 2026 Feb 8;65(12):e20411. doi: 10.1002/anie.202520411 (PMC12991037; doi:10.1002/anie.202520411)
Supplement: Supplementary file 1 — Supporting File 1: anie71352‐sup‐0001‐SuppMat.docx. [file ANIE-65-e20411-s001.docx]

Supporting Information

Distance-Dependent Energy Transfer between Organic Fluorophores and Single-Walled Carbon Nanotubes

Izabela Kamińska,^[a,b]^ Justus T. Metternich,^[c,d]^ Alan M. Szalai,^[e]^ Carolin Smidoda,^[a]^ Sayantani Chakraborty,^[f]^ Lela Vukovic,^[c,f,g,h]^ Sebastian Kruss,*^[c,d]^ Philip Tinnefeld*^[a]^

[a] Dr. Izabela Kamińska, Carolin Smidoda, Prof. Philip Tinnefeld
Department of Chemistry and Center for NanoScience
Ludwig Maximilian University of Munich
Butenandtstraße 5-13
81377 Munich, Germany
E-mail: philip.tinnefeld@lmu.de

[b] Dr. Izabela Kamińska
Institute of Physical Chemistry of the Polish Academy of Sciences
Kasprzaka 44/52
01-224 Warsaw, Poland

[c] Dr. Justus T. Metternich, Prof. Lela Vukovic, Prof. Sebastian Kruss
Department of Chemistry and Biochemistry
Ruhr-University Bochum
Universitätsstrasse 150
44801 Bochum, Germany
E-mail: sebastian.kruss@rub.de

[d] Dr. Justus T. Metternich, Prof. Sebastian Kruss
Biomedical Nanosensors
Fraunhofer Institute for Microelectronic Circuits and Systems
Finkenstrasse 61
47057 Duisburg, Germany

[e] Dr. Alan M. Szalai
Centro de Investigaciones en Bionanociencias (CIBION)
Consejo Nacional de Investigaciones Científicas y Técnicas (CONICET)
Godoy Cruz 2390, C1425FQD Ciudad Autónoma de Buenos Aires, Argentina

[f] Sayantani Chakraborty, Prof. Lela Vukovic
Department of Chemistry and Biochemistry
University of Texas El Paso
El Paso, Texas 79968, United States

[g] Prof. Lela Vukovic
Computational Science Program
University of Texas El Paso
El Paso, Texas 79968, United States

[h] Prof. Lela Vukovic
Bioinformatics Program
University of Texas El Paso
El Paso, Texas 79968, United States

**Table of Contents**

[1. Materials and Methods 4](#_Toc213996127)

[1.1. Buffers 4](#_Toc213996128)

[1.2. SWCNT surface modification 4](#_Toc213996129)

[1.3. Sample preparation 5](#_Toc213996130)

[2. Imaging and Analysis 6](#_Toc213996131)

[2.1. NIR-Spectroscopy 6](#_Toc213996132)

[2.2. Fluorescence measurements 8](#_Toc213996133)

[2.3. Fluorescence lifetime analysis 8](#_Toc213996134)

[3. Computational Methods 13](#_Toc213996135)

# Materials and Methods

## Buffers

Unless stated otherwise, all experiments were conducted with commercially available (6,5)-enriched single wall carbon nanotubes (SWCNTs) from Sigma Aldrich / Chasm Advanced Materials, Signis® SG65i, CoMoCAT™, ≥ 95% carbon basis (≥ 95% as carbon nanotubes, ≈ 41% as (6,5)-SWCNTs), 0.78 nm average diameter) in 1× phosphate buffered saline buffer, pH 7.4 (PBS; Carl Roth) with 10 mM MgCl_2_ (Sigma Aldrich). Water used for the preparation of buffers was of ultrapure (type 1) grade. Single-stranded DNA (ssDNA) was purchased from Biomers or Ella Biotech.

## SWCNT surface modification

The surface modification of SWCNTs followed a modification of a previously published protocol (Figure S1).^[1]^


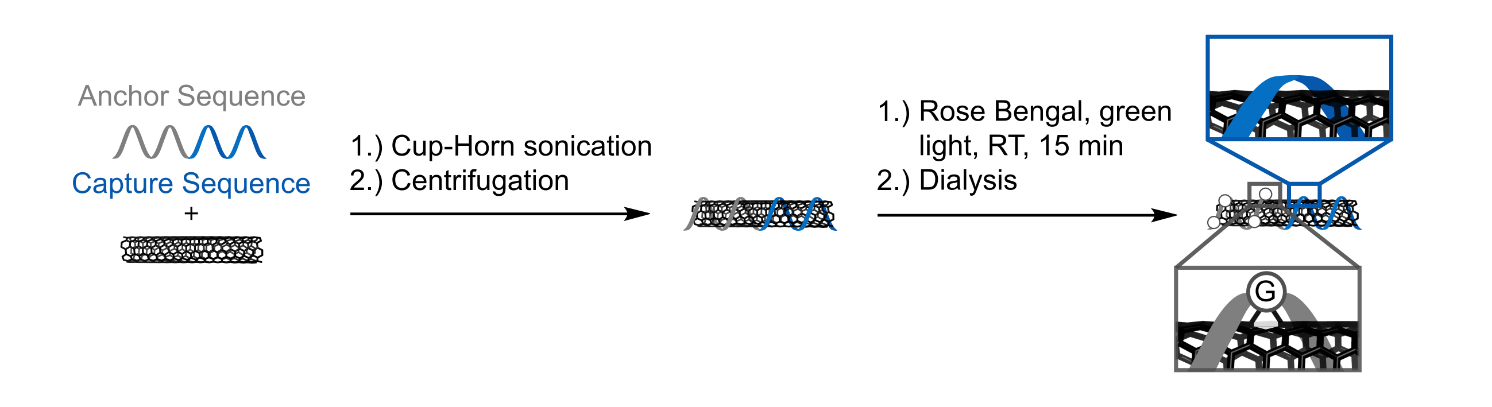


**Figure S1.** Preparation of SWCNTs. SWCNTs were non-covalently functionalized with ssDNA containing an anchor (with guanine) and a capture sequence (without guanine). Guanine defects were introduced via ^1^O_2_ that was generated by irradiation of Rose Bengal under ambient atmosphere. Note that the size of the structures is not to scale.

In short, ssDNA (200 μL, 100 µM in PBS, Table S1) was mixed with SWCNTs (100 μL, 2 mg/mL in PBS). The samples were sonicated (Fisher-brand^TM^, Q705 Sonicator with a 14 cm cup-horn, 1% amplitude, 20 min pulsed: 9 s on, 1 s off). The resulting suspension was centrifuged (2×, 21000 g, 30 min). The pellet of each centrifugation step was discarded, and the respective supernatant was transferred into a different reaction vessel and used for covalent attachment of guanines in the anchor sequences with the SWCNTs.

The DNA-functionalized SWCNTs (187.9 μL, 10.6 nM in PBS) were mixed with Rose Bengal (12.1 μL, 330 μM in H_2_O, Sigma-Aldrich) in a 96-microwell plate and irradiated (Analytical Sales Lumidox 96-Well Green LED Array + LED Controller, 25 mA, ≈ 527 nm). After 10 min, air was bubbled through the mixtures and the respective wells were irradiated for an additional 5 min. Free DNA and Rose Bengal were removed via dialysis (Spectrum Laboratories SpectraPor membranes, molecular weight cut-off: 300 kDa) for five days with buffer changes twice a day. The suspensions were briefly sonicated for redispersion (35 % amplitude, 20 s), centrifuged (21000 g, 20 min) and the supernatant was used for further studies.

Absorption (Figure S3) Excitation-emission spectra (Figure S5) were recorded to ensure that all SWCNT-DNA hybrids possess comparable photophysical characteristics.

**Table S1.** Sequences used for the functionalization of SWCNTs.

| Name | Sequence |
| --- | --- |
| (GT)_15_-S0-12b | GTGTGTGTGTGTGTGTGTGTGTGTGTGTGTCTACATCACAAA |
| (GT)_15_-S0-15b | GTGTGTGTGTGTGTGTGTGTGTGTGTGTGTCACCTACATCACAAA |
| (GT)_15_-S0-18b | GTGTGTGTGTGTGTGTGTGTGTGTGTGTGTTACCACCTACATCACAAA |
| (GT)_15_-S0-20b | GTGTGTGTGTGTGTGTGTGTGTGTGTGTGTTCTACCACCTACATCACAAA |
| (GT)_15_-S0-24b | GTGTGTGTGTGTGTGTGTGTGTGTGTGTGTTTCCTCTACCACCTACATCACAAA |

## Sample preparation

For duplex formation, the DNA-functionalized SWCNT suspension (100 μL) was mixed with 100 μL of the imager S1 oligonucleotide labeled with ATTO542 or ATTO643 (5 nM in 1× PBS supplemented with 10 mM MgCl₂) (Table S2). The concentration of the SWCNT suspension was 3–5 nM before mixing (final 1.5–2.5 nM after the 1:1 mix). Thus the final imager:SWCNT ratio during hybridization was ~1–1.7:1, i.e., sub-stoichiometric with respect to the effective number of accessible capture strands per nanotube (Figure S2). To ensure single occupancy in the single-molecule dataset, we only analyzed emitters that exhibited a single photobleaching step. The mixture was incubated for 2 h at room temperature and stored overnight at 4 °C. Glass coverslips were sequentially cleaned by sonication in isopropanol and Milli-Q water (15 min each), and an incubation chamber was affixed to the coverslip. The SWCNT+ATTO542/ATTO643 suspension was diluted 1:1 with 1× PBS containing 10 mM MgCl₂, injected into the chamber, incubated for 1 h, and washed three times with 1× PBS supplemented with 10 mM MgCl₂.


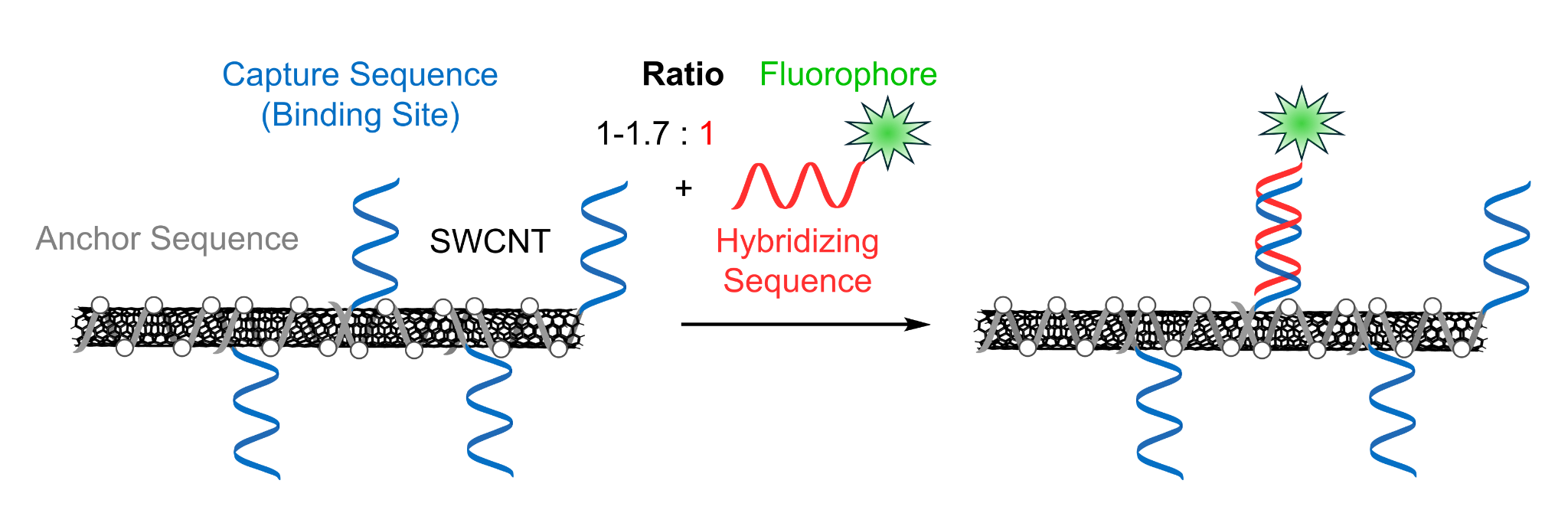


**Figure S2.** Single occupancy binding of dye-labeled imager strands on SWCNTs via sub-stoichiometric mixing with respect to the effective number of binding sites.

**Table S2.** Imager sequences.

| Name | Sequence |
| --- | --- |
| S1 | TTTGTGATGTAGGTGGTAGAGGAA |
| S1_ATTO542 | [ATTO542]-TTTGTGATGTAGGTGGTAGAGGAA |
| S1_ATTO643 | [ATTO643]-TTTGTGATGTAGGTGGTAGAGGAA |

# Imaging and Analysis

## NIR-Spectroscopy

Absorption spectra of SWCNTs were recorded at room temperature with a JASCO V-670 device in a 10 mm path cuvette (Brand UV cuvette micro, Figure S3).


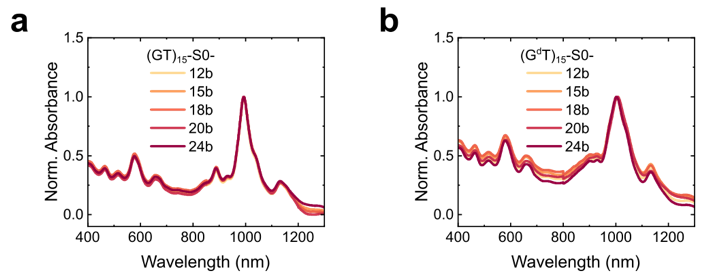


**Figure S3.** Normalized absorption spectra of SWCNTs (a) before and (b) after the introduction of guanine defects.

**
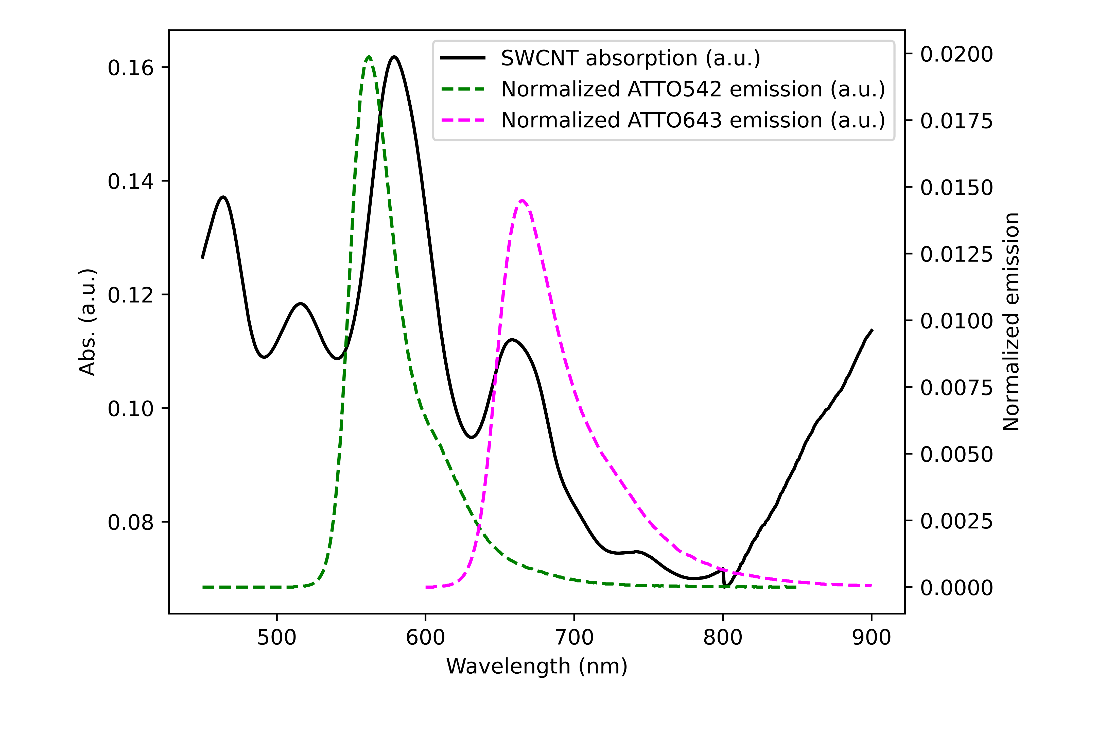
**

**Figure S4.** Normalized absorption of (G^d^T)_15_-S0-12b (solid black line) and normalized emission spectra of ATTO542 and ATTO643 (green and magenta dashed lines, respectively).

NIR-Fluorescence spectra were recorded on a custom-built setup at 22°C. Briefly, a diode pumped solid state laser (Laser Quantum gem-561, 561 nm) was used for the excitation of the sample with a 20x objective (Olympus LCPLN-IR, NA = 0.45) on a microscope (Olympus IX73) connected to a spectrograph (Andor Technology Shamrock 193i equipped with an Andor iDus InGaAs 491 detector). 2D Excitation-Emission spectra were recorded on the same set-up. For excitation, a monochromator (Quantum Design GmbH MSH-

150) equipped with a xenon arc lamp and a diffraction grating (Quantum Design GmbH, MSG-T-1200-500, Line density: 1200 g/mm, Blaze wavelength: 500 nm) was used. The sample was excited between 400 nm and 700 nm in 5 nm steps and the fluorescence at each excitation wavelength was recorded on the spectrograph. All fluorescence spectra were background corrected in the spectrometer software using a spectrum of the buffer (Figure S5).


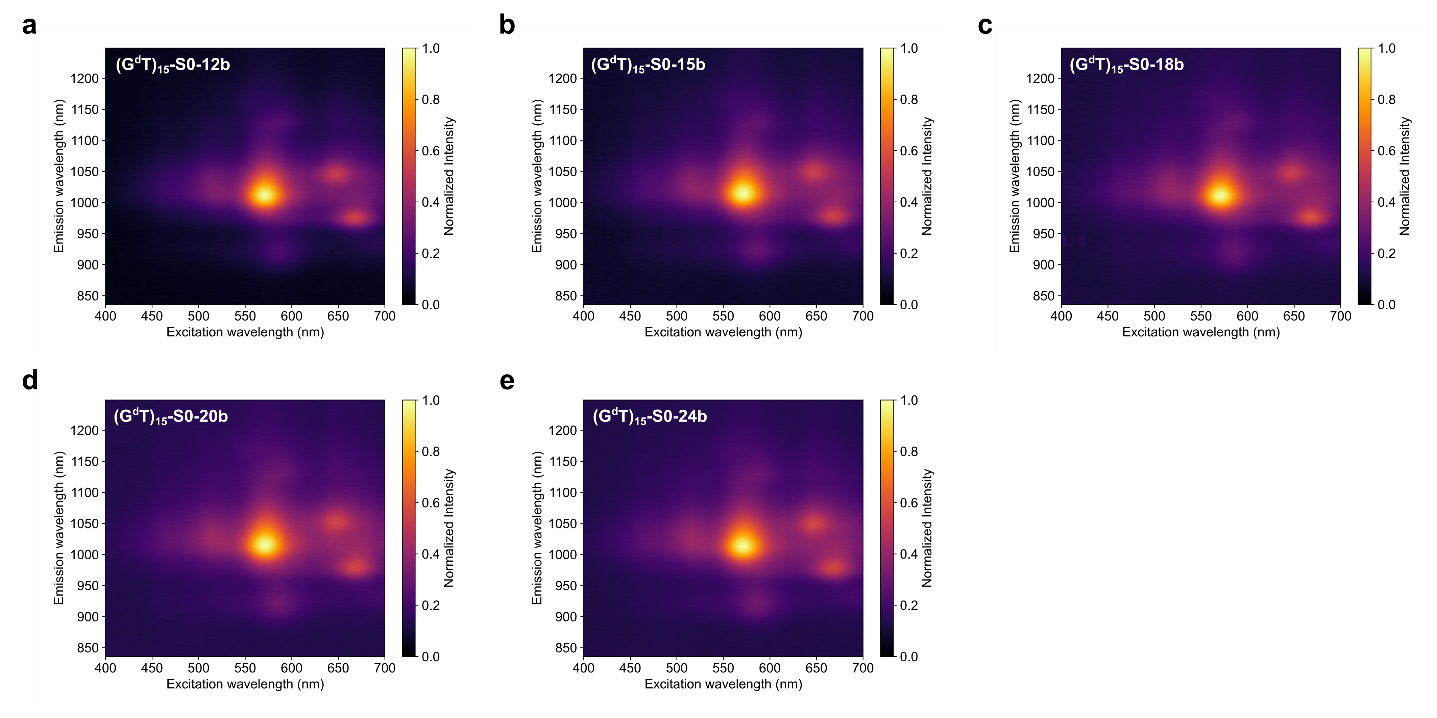


**Figure S5.** 2D Excitation-Emission spectra of different DNA-SWCNT samples. DNA sequences are shown in the upper left of each spectrum.

To test the influence of hybridization and fluorescent dyes, we recorded the NIR fluorescence response of SWCNTs. The NIR fluorescence of functionalized SWCNTs (198 μL, 0.1 nM in PBS with 10 mM MgCl_2_) was recorded in a microwell plate prior, as well as at the defined timepoints after the addition of the respective oligonucleotide (2 μL, 100 μM) or buffer (Figure S6 and S7).


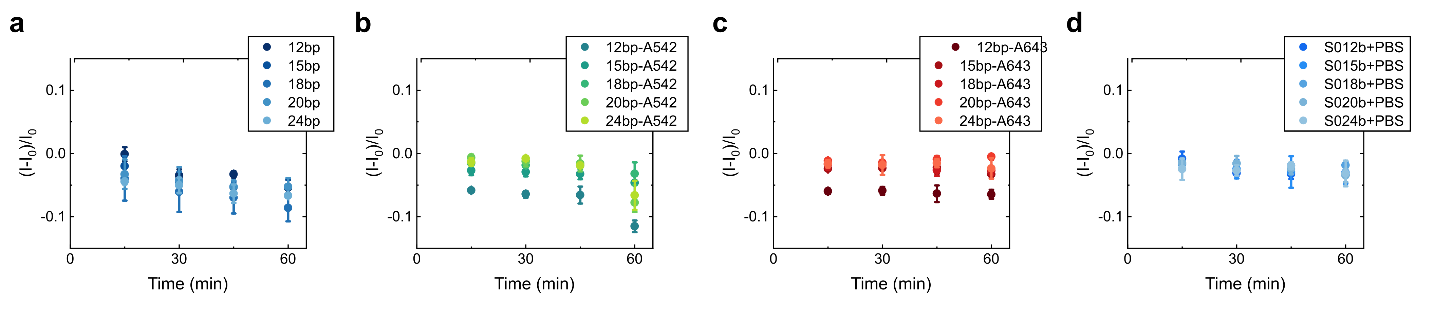


**Figure S6.** NIR response of SWCNTs after the addition of (a) complementary DNA, (b-c) labelled complementary DNA (imager S1 sequence), or (d) buffer (n=3, mean ± SD).


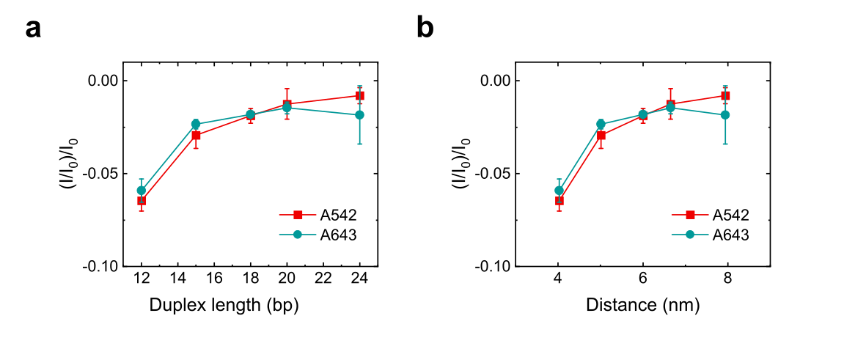


**Figure S7.** Distance dependent NIR response of SWCNTs to ATTO542 and ATTO643. SWCNTs were incubated with an imager S1 sequence for 30 min. Note that data is shown in Figure S6 (n=3, mean ± SD).

## Fluorescence measurements

FLIM images and single-molecule fluorescence time traces were performed on a custom-made confocal microscope. The setup is based on an Olympus IX-71 inverted microscope, and samples were excited using pulsed diode lasers at 532 nm or 636 nm (LDH-P-FA-530B and LDH-D-C-640; PicoQuant GmbH, Germany) operating at 40 MHz (PDL 828 “Sepia II” with oscillator module SOM 828, PicoQuant GmbH, Germany). Both laser beams were coupled into a single-mode fiber (P3-488PM-FC, Thorlabs GmbH, Germany) to produce a Gaussian beam profile and ensure precise spatial overlap of the excitation paths. Circular polarization was generated using a linear polarizer (LPVISE100-A, Thorlabs GmbH, Germany) in combination with a quarter-wave plate (AQWP05M-600, Thorlabs GmbH). The light was focused to a diffraction-limited spot with an oil-immersion objective (UPLSAPO100XO, NA 1.40, Olympus Deutschland GmbH, Germany). Sample positioning was controlled by a piezoelectric stage (P 517.3CD, Physik Instrumente GmbH & Co. KG, Germany) driven by a controller (E-727.3CDA, Physik Instrumente GmbH & Co. KG, Germany).

Emission light was separated from the excitation beams using a dichroic beamsplitter (zt532/640rpc, Chroma Technology Corporation, USA) and passed through a 50 µm pinhole (Thorlabs GmbH, Germany). After the pinhole, emission was split into green (Brightline HC582/75, AHF, Germany; RazorEdge LP 532, IDEX Health & Science, LLC, USA) and red (SP 750, AHF, Germany; RazorEdge LP 647, IDEX Health & Science, LLC, USA) detection channels using a second dichroic beamsplitter (640 LPXR, Chroma Technology Corporation, USA). Signals were detected with avalanche photodiodes (APDs) (SPCM-AQRH-14-TR, Excelitas Technologies Corporation, USA) and recorded via a time-correlated single photon counting module (HydraHarp400, PicoQuant GmbH, Germany). System control and data acquisition were performed using SymPhoTime64 (PicoQuant GmbH, Germany).

The laser powers at the objective were set to 4 µW for excitation at both wavelengths. The instrument response function (IRF) was measured at each excitation wavelength focusing the excitation light directly on a coverslip (without fluorescent sample mounted on it) and directly detecting the reflected light on the APDs.

## Fluorescence lifetime analysis

FLIM images and fluorescence lifetime traces (see Figure S8-S11 for exemplary time traces) were processed with a custom Python script.^[2]^ For FLIM images, the lifetime was obtained in each pixel using a least-squares monoexponential tail-fit, without IRF deconvolution. For time traces, the lifetime was obtained using a Maximum Likelihood Estimation (MLE), where a monoexponential model was considered, and the experimental IRF was considered for deconvolution.

The fit of the lifetime populations was done using Gaussian Mixture Model, varying the number of subpopulations, constraining the width (sigma) of each subpopulation to the (0.05-0.5) ns range. To use the minimum number of subpopulations and avoid overfitting, we minimized the AIC (Akaike Information Criterion), computing this value for *n* = 1 to 5 Gaussians for all cases. We kept the *n* corresponding to the minimum AIC, unless there was a fit with smaller *n* and a similar AIC value (up to 5% larger compared to the minimum case).

**Table S3.** Fluorescence lifetime and quenching efficiency of less-quenched populations.

| System | Fluorescence lifetime of less-quenched population (mean) [ns] | Fluorescence lifetime of less-quenched population (std) [ns] | Quenching efficiency |
| --- | --- | --- | --- |
| 12bp-ATTO542 | 0.17 | 0.34 | 0.94 |
| 12bp-ATTO643 | 0.22 | 0.12 | 0.93 |
| 15bp-ATTO542 | 0.75 | 0.37 | 0.75 |
| 15bp-ATTO643 | 0.79 | 0.41 | 0.78 |
| 18bp-ATTO542 | 1.37 | 0.5 | 0.53 |
| 18bp-ATTO643 | 1.57 | 0.40 | 0.56 |
| 20bp-ATTO542 | 1.74 | 0.27 | 0.42 |
| 20bp-ATTO643 | 2.24 | 0.27 | 0.37 |
| 24bp-ATTO542 | 1.94 | 0.5 | 0.35 |
| 24bp-ATTO643 | 2.48 | 0.34 | 0.3 |

#

**
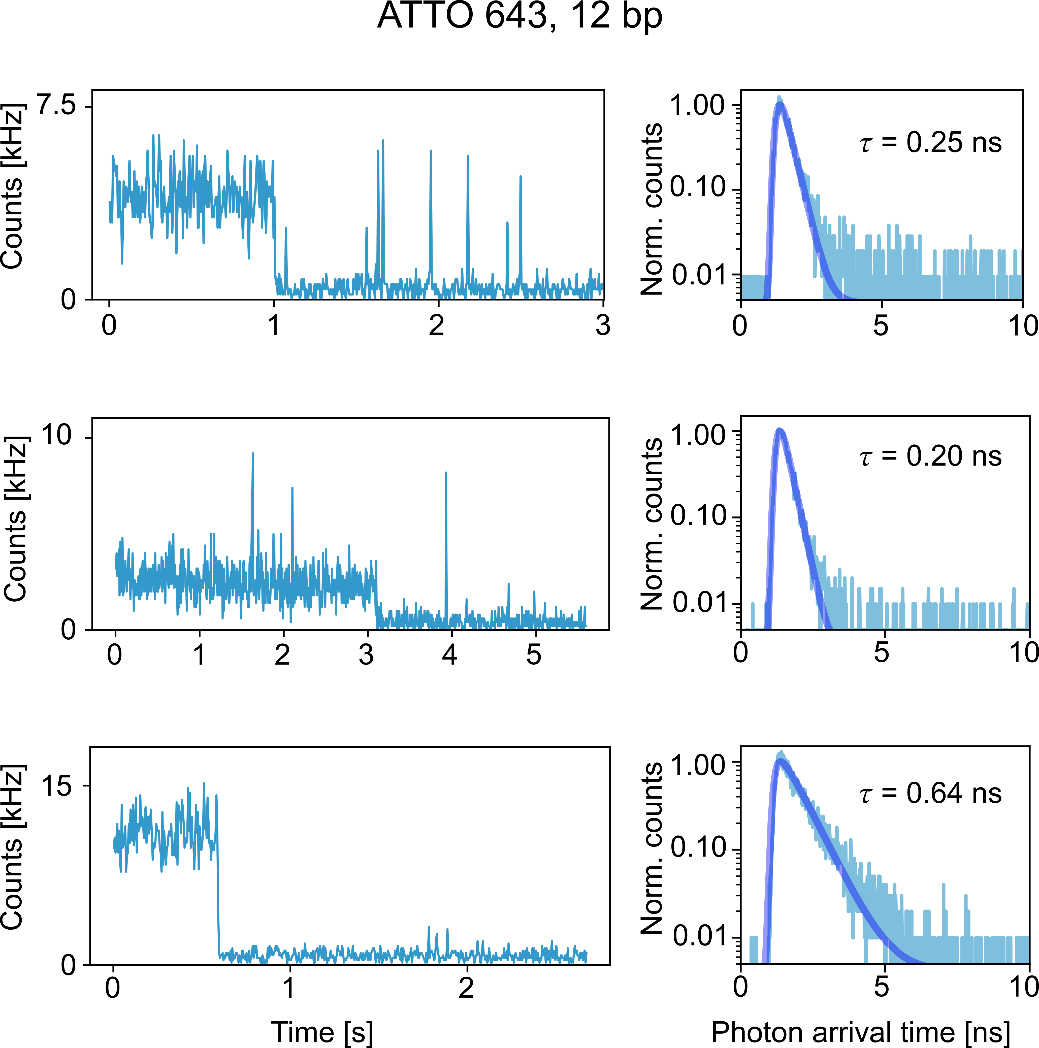
**

**Figure S8.** Additional exemplary time traces and their corresponding fluorescence decay curves for 12bp systems functionalized with ATTO643.

**
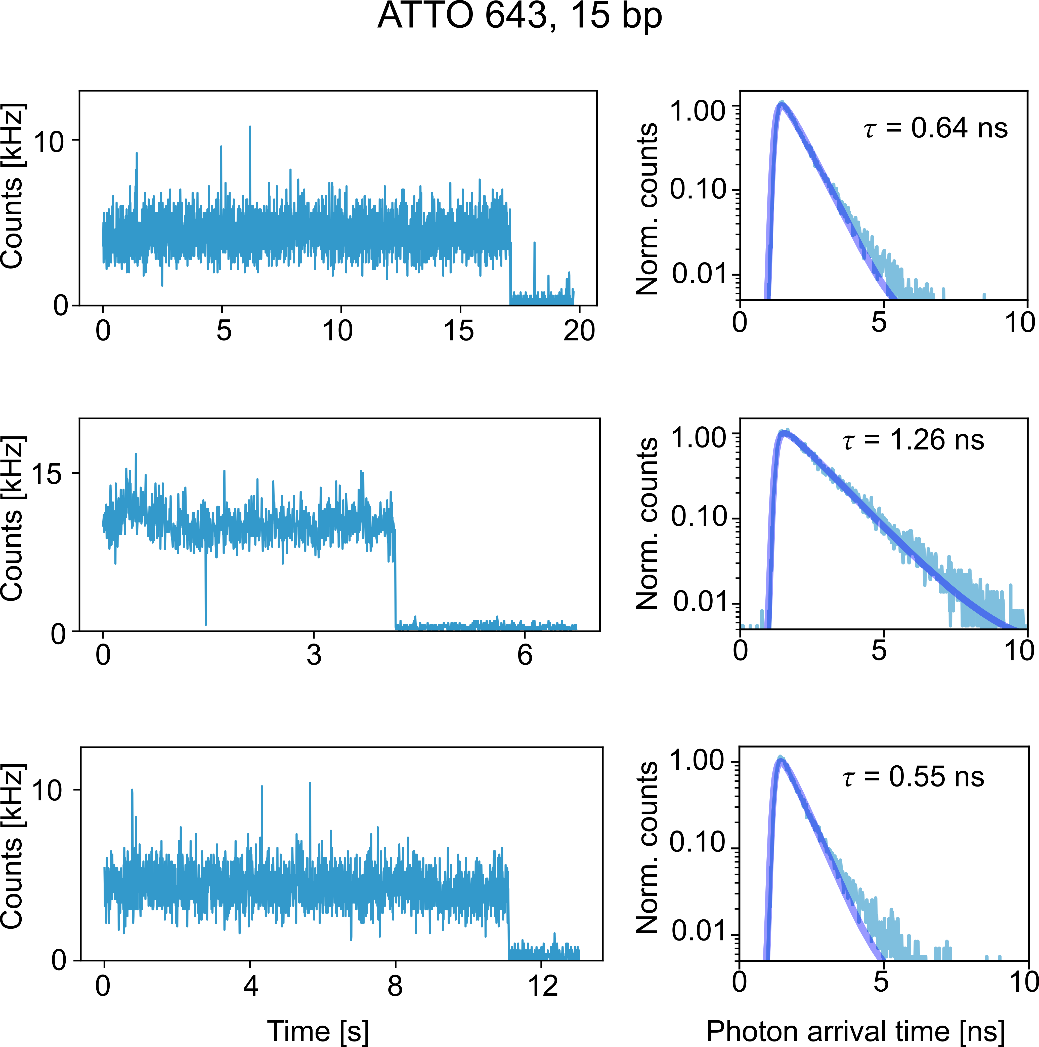
**

**Figure S9.** Additional exemplary time traces and their corresponding fluorescence decay curves for 15bp systems functionalized with ATTO643.

**
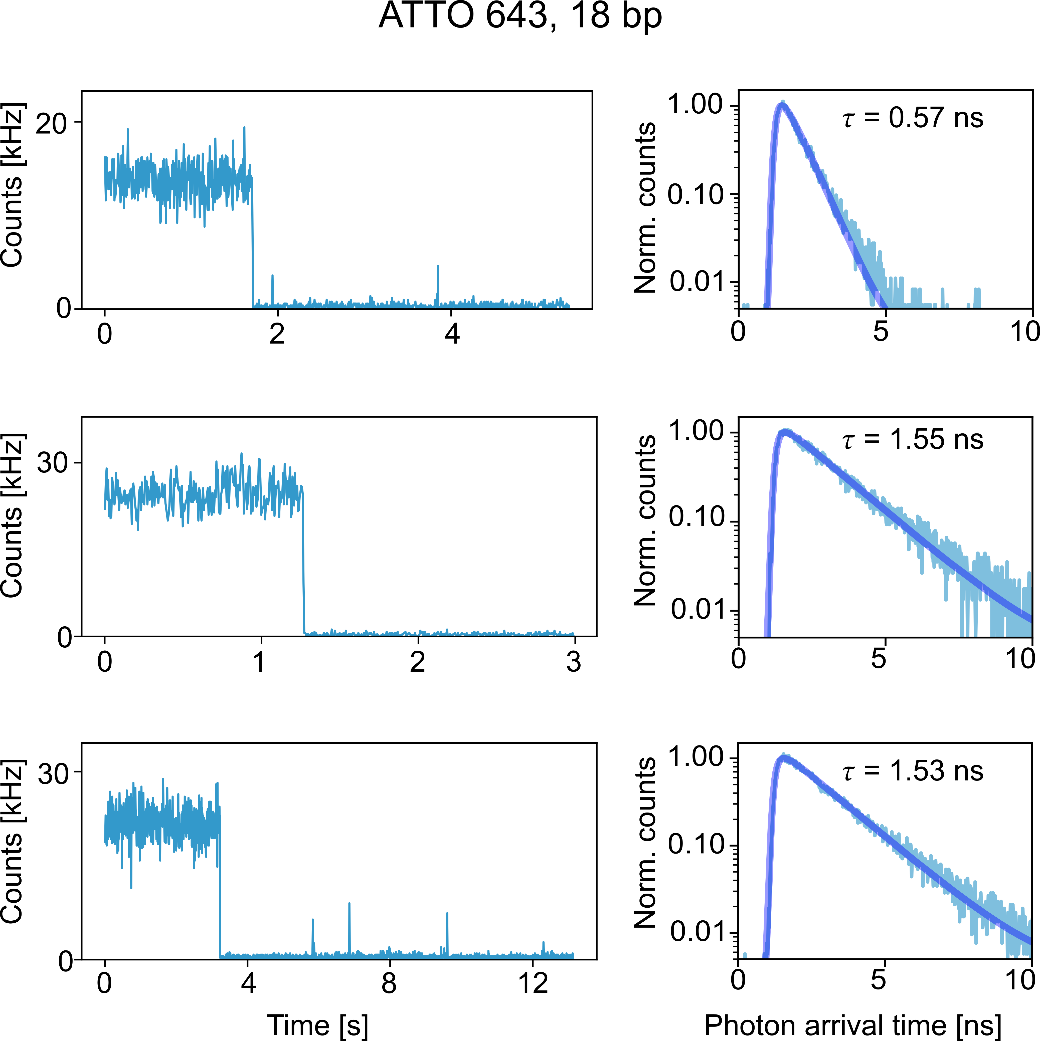
**

**Figure S10.** Additional exemplary time traces and their corresponding fluorescence decay curves for 18bp systems functionalized with ATTO643.

**
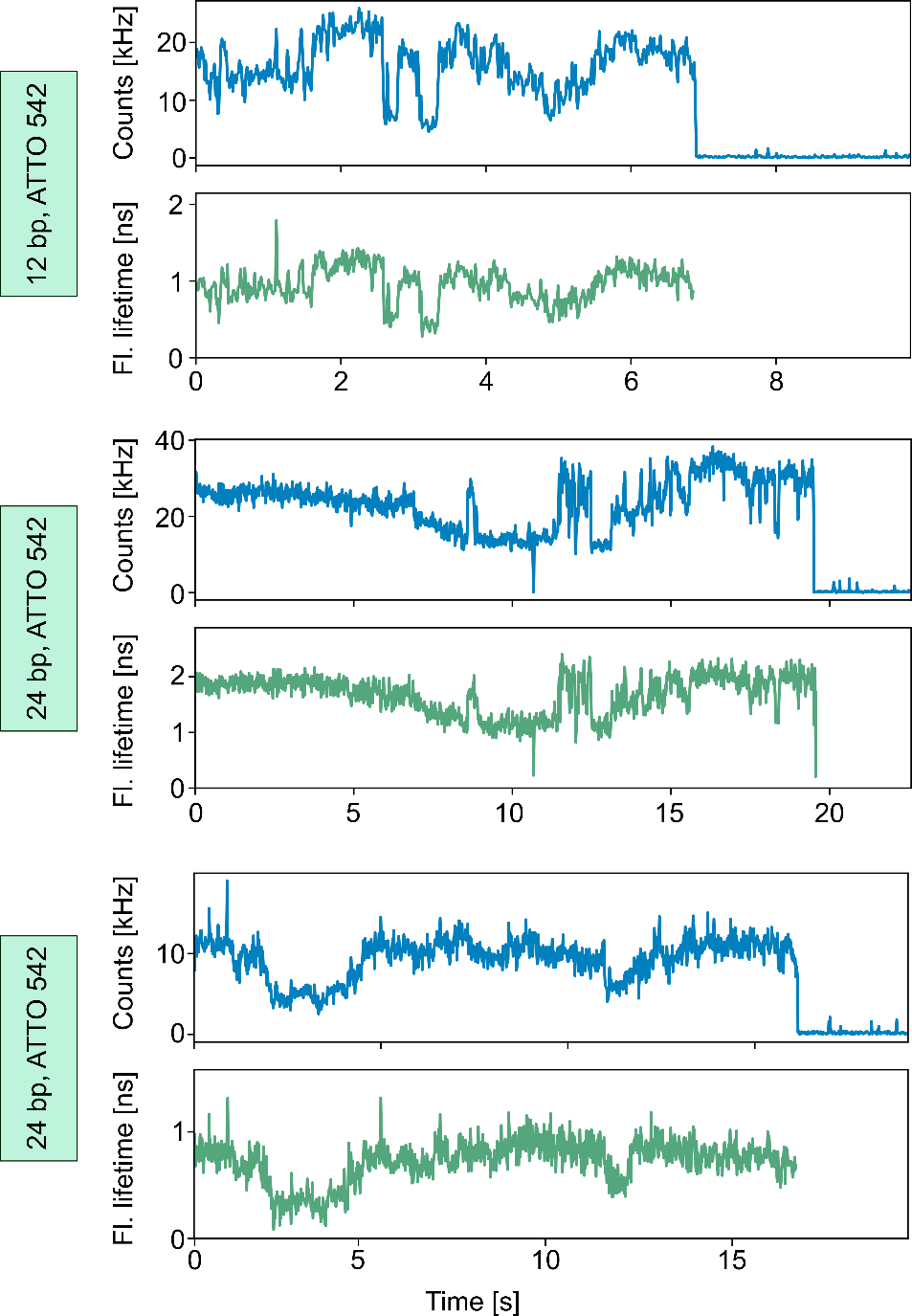
**

**Figure S11.** Example time traces for 12bp and 24bp systems functionalized with ATTO542, showcasing fluorescence lifetime correlated intensity fluctuations that are reflecting distance changes of the dye to the SWCNT.

Independent measurements of dye-strands adsorbed on glass coverslips (Figure S12-S13) were performed to substantiate the assignment of non-quenched fractions in Figure 1d to dye-labeled DNA that was not binding to SWCNTs, but unspecifically attached to the coverslip.

**
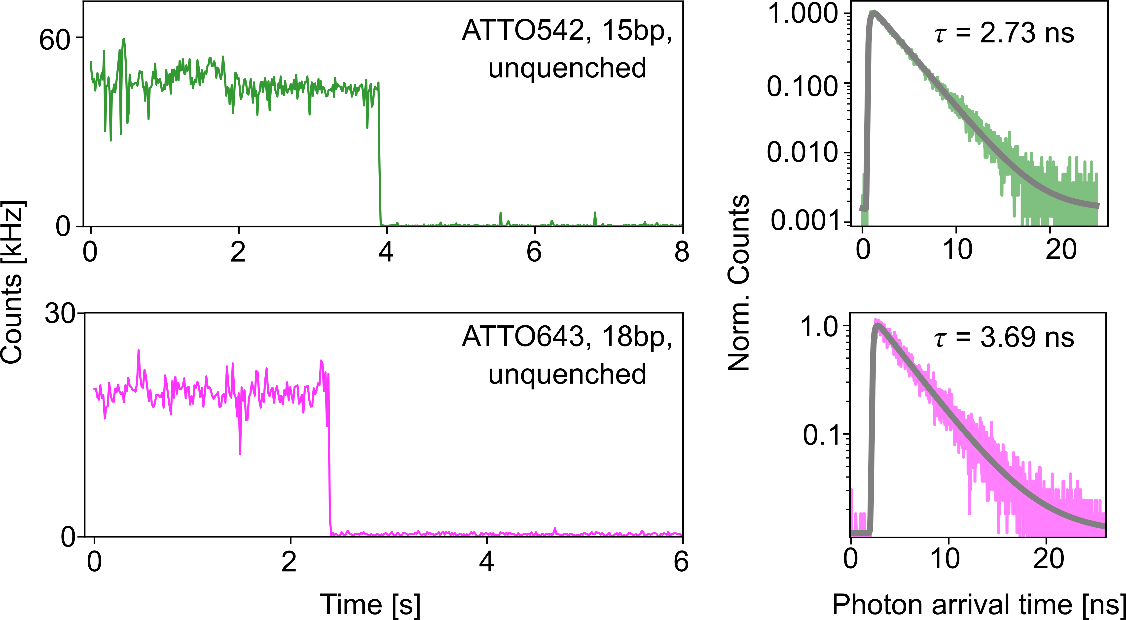
**

**Figure S12.** Example time traces of unquenched fluorophores and their respective fluorescence decay curves.

**
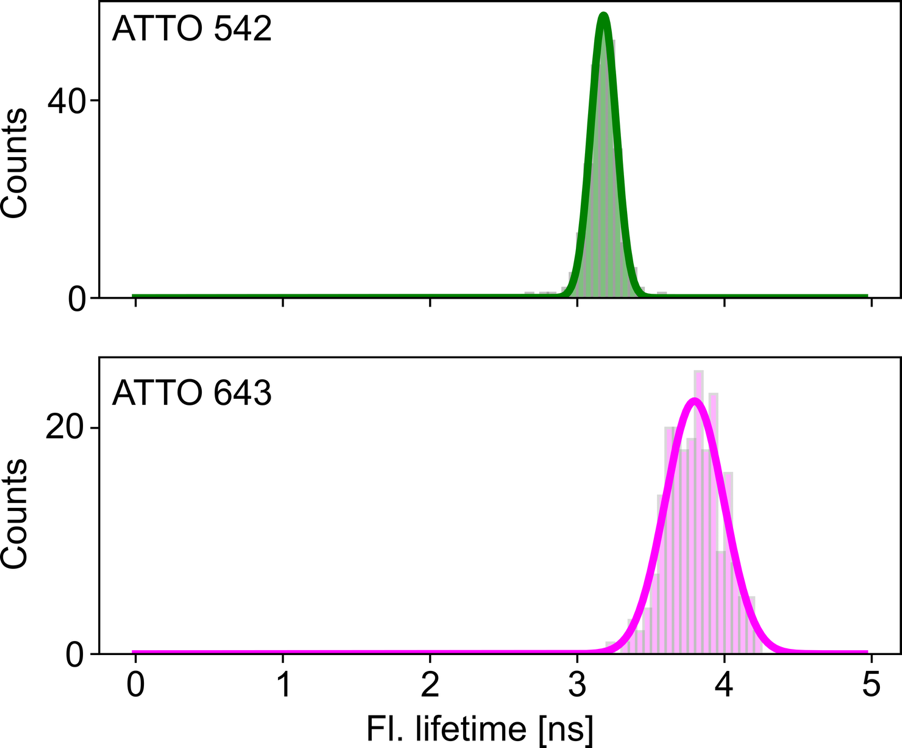
**

**Figure S13.** Histograms of fluorescence lifetime values obtained for ssDNA labeled with ATTO542 (top) and ATTO643 (bottom) adsorbed on glass coverslips.

# Computational Methods

Molecular Dynamics Simulations. We modeled five DNA-SWCNT systems using classical all-atom MD simulations. Each system consisted of an 8 nm-long (6,5) SWCNT wrapped by an anchor DNA strand with the sequence (GTG^d^T)_7_GT, where G^d^ refers to guanine residues covalently attached to the SWCNT via guanine defect chemistry.^[1, 3-5]^ The anchor continued into the capture strand, which varied in sequence and length (Table S4) and formed a duplex through base pairing with a 24-nucleotide-long complementary strand, which remained constant across all systems. The five modeled systems differed in the length of the formed duplex, namely, 12, 15, 18, 20, or 24 base pairs. For duplexes shorter than 24bp, the complementary strand had 3′-end single stranded overhangs of 12, 9, 6, or 4 nucleotides. The systems were named according to duplex length: 12bp, 15bp, 18bp, 20bp, and 24bp.

**Table S4.** Sequences in the simulated systems.

| System | Capture sequence (3’-5’) | Hybridizing sequence (5’-3’) | Overhang sequence (5’-3’) |
| --- | --- | --- | --- |
| 12bp | CTACATCACAAA | TTTGTGATGTAG | 5’-GTGGTAGAGGAA |
| 15bp | CACCTACATCACAAA | TTTGTGATGTAGGTG | GTAGAGGAA |
| 18bp | TACCACCTACATCACAAA | TTTGTGATGTAGGTGGTA | GAGGAA |
| 20bp | TCTACCACCTACATCACAAA | TTTGTGATGTAGGTGGTAGA | GGAA |
| 24bp | TTCCTCTACCACCTACATCACAAA | TTTGTGATGTAGGTGGTAGAGGAA | N/A |

A stepwise procedure was used to construct each DNA-SWCNT conjugate, starting with the 12bp system. First, the (6,5)-SWCNT was generated using the Nanotube Builder plugin in VMD.^[6]^ The initial (GT)_15_ anchor strand was modeled in PyMOL^[7]^ and wrapped helically around the SWCNT surface. Next, duplex structures were generated using the 3DNA web server.^[8]^ One strand of the 12-nt-long duplex was positioned so its 5′-end P atom aligned with the 3′-end O3′ atom of the 30th thymine in the (GT)_15_ anchor strand. A covalent bond was then formed between the two atoms using the *psfgen* plugin in VMD.

For the 12bp system with the longest complementary strand overhang, the overhang was created by extracting a 12nt sequence from the pre-built (GT)_15_ strand, translating and rotating it to the desired position on the SWCNT, and mutating the bases to match the complementary strand sequence. This ensured that the 5′-end of the overhang was also positioned near the 3′-end of the 30th thymine in the (GT)_15_ anchor strand. To bring the 3′ of the other duplex strand (O3′) near the P atom of the 5′-end residue of the complementary strand overhang, we performed a steered MD simulation in explicit TIP3P water with neutralizing Na⁺ ions. The two DNA atoms described above were brought to within 3 Å of each other using the collective variable distance constraints, with a harmonic potential with force constant of 50 kcal·mol^-1^Å^-2^, over a 2 ns-long MD simulation. During this simulation, the (GT)_15_ part and the SWCNT atoms were restrained with a harmonic potential of 1 kcal mol^-1^Å^-2^. When the atoms reached the target distance after 2 ns, a covalent bond between the second duplex strand and the overhang was introduced into the final structure using *psfgen* plugin, thus completing the structure of the intact complementary strand. All the systems were described with CHARMM36 force field parameters,^[9-12]^ as they have been successfully used in modeling interactions between ssDNA molecules and SWCNTs in previous studies.^[13-17]^ The simulations were performed with NAMD2.13 package^[18]^ using Langevin dynamics in the NpT ensemble, a Langevin constant $\gamma_{Lang}$of 1.0 ps^-1^, pressure of 1 bar, and a temperature of 298 K. The integration time step was 2 fs, and Coulomb and van der Waals non-bonded interactions were evaluated every one- and two-time steps, respectively, for all atoms within a 12 Å cutoff distance. The particle-mesh Ewald (PME) method^[19]^ was used to evaluate the long-range Coulomb interactions, with periodic boundary conditions applied in all directions. Prior to the steered MD, the system was minimized for 5,000 steps, and the solvent molecules were equilibrated for 0.1 ns, while keeping all the other atoms in the system restrained to their original positions with the harmonic force and the force constant of 1 kcal mol^-1^Å^-2^.

In the final step, seven guanine defects were introduced into the (GT)_15_ anchor strand using a custom CHARMM force field patch, based on the guanine defect chemistry proposed in^[5]^. Parameters for a new guanine defect residue were initially generated for a model system consisting of a guanine nucleotide covalently attached to a SWCNT segment using the CGENFF web server (https://cgenff.com), based on CHARMM force field. These parameters were then used to manually create a CHARMM-compatible patch. This patch creates a covalent bond between the C8 atom of the guanine residue and specified carbon atoms on the SWCNT, adjust the partial charges in the modified guanine and the SWCNT atoms, and make the overall charge of the whole guanine defect site -1. The structure file (gd.str) containing the patch parameters is included in the Supplementary Information. This patch was applied to create guanine defects at positions 3, 7, 11, 15, 19, 23, and 27 within the (GT)_15_ segment of the anchor strand. A representative final structure of one of the final systems (12bp) is shown in Figure 2a.

The final 12bp system was solvated in TIP3P water and 0.1 M NaCl. Atomistic simulations were performed using NAMD2.13 and the above-described settings, with all the SWCNT atoms restrained to their original positions using harmonic restraints (spring constant of 1 kcal (mol·Å^2^)^-1^). The system was first minimized for 5,000 steps, during which the backbones of the (GTG^d^T)_7_GT segment and the duplex region were restrained using a spring constant of 5 kcal (mol·Å^2^)^-1^. Following minimization, the overhang segment of the complementary strand was equilibrated for 50 ns, keeping the other atoms under the same restraint conditions. Next, the duplex region was equilibrated for 10 ns, while harmonic restraints 5 kcal (mol·Å^2^)^-1^ were applied to the backbone atoms of both the (GTG^d^T)_7_GT sequence and the overhang segment.

The last frame of the equilibrated 12bp system was used to prepare 15bp, 18bp and 20bp systems. New duplexes of 15, 18, and 20 base pairs were generated using the 3DNA 2.0 web server and aligned with the 12nt duplex present in the relaxed 12bp structure. The overhang segment was then shortened and mutated for each system, and covalent bonds were created using the *psfgen* plugin to connect the duplexes to the remaining segments of anchor and overhang sequences. Each resulting system was solvated in TIP3P water and 0.1 M NaCl, minimized for 5,000 steps and equilibrated for 10 ns. During equilibration, the SWCNT atoms were restrained using a spring constant of 10 kcal (mol·Å^2^)^-1^, and the backbone atoms of the (GTG^d^T)_7_GT and overhang strands were restrained with a spring constant of 5 kcal (mol·Å^2^)^-1^

The 24bp system was prepared by positioning the 5’-end P atom of one duplex strand near the O3’ atom of the 3’-end thymine of the (GT)_15_ strand, and creating the covalent bond between these atoms using the *psfgen* plugin. The CHARMM patch file was then used to introduce seven guanine defects. The system was solvated in TIP3P water with 0.1 M NaCl, minimized for 5,000 steps, and briefly equilibrated for 0.1 ns with harmonic restraints of 10 kcal·mol^-1^·Å^-2^ and 1 kcal·mol^-1^·Å^-2^ applied to all SWCNT atoms and all DNA backbone atoms, respectively.

Finally, production MD runs with harmonic restraint applied on SWCNT atoms, with spring constant of 1 kcal (mol·Å)^−1^, were performed for all systems for 400ns. The final system sizes are reported in Table S5.

**Table S5.** Summary of simulations performed.

| System | Total number of atoms | Simulation time (ns) |
| --- | --- | --- |
| 12bp | 118,790 | 400 |
| 15bp | 111,868 | 400 |
| 18bp | 114,597 | 400 |
| 20bp | 143,908 | 409.5 |
| 24bp | 181,032 | 397.7 |

*Angle calculations.* To analyze the orientations that the duplexes have with respect to the SWCNT surface, we calculated the angle ($\theta$) between a vector that follows the long axis of the duplex $(\vec{d})$and the vector that runs parallel with the SWCNT axis ($\vec{z})$ (Figure 2b inset):

$\theta=\cos^{-1} \frac{\vec{d}\cdot\vec{z}}{\left| \vec{d} \right|\left| \vec{z} \right|}$ (1)

The vector $\vec{d}$that follows the long axis of the duplex joins the center of mass (COM) of the bottom three residues of the duplex in capture and hybridizing sequences, nearest to the SWCNT surface, and the top three residues at the end of the duplex, starting from the second to top residue, on both the capture and the hybridizing sequences:

*Distance calculations.* To calculate the distance of the farthest end of the duplex from the SWCNT surface, we calculated distances between the centers of mass of the 5′-end residue of the hybridizing strand (the duplex tip where the dye is attached in the experiment), and the SWCNT surface at time *t*:

$d\left( t \right)=r_{5^{'}-residue}(t)-r_{SWNT}$ (2)

where $r_{5^{'}-residue}(t)$ is the radial distance of the center of mass of the 5′-end residue of the hybridizing strand at time *t*, defined in the cylindrical coordinate system, and $r_{SWNT}$ is the radius of the (6,5)-SWCNT.

To estimate duplex lengths, we use the average rise per base in duplex DNA of 0.34 nm multiplied by the number of base pairs, resulting in 4.08 nm, 5.1 nm, 6.12 nm, 6.8 nm and 8.16 nm long duplexes with 12, 15, 18, 20, and 24 base pairs.

**Table S6.** Percentage of simulation time without base pairing of the 5′-end nucleotide in the hybridizing strand of the duplex. The percentage was based on calculating the number frames in which the atoms of two bases at the farthest end of the capture sequence and the hybridizing sequence are further than 5 Å away from each other.

| System | % of time |
| --- | --- |
| 12bp | 87.7 |
| 15bp | 86.6 |
| 18bp | 93.01 |
| 20bp | 83.9 |
| 24bp | 85.1 |


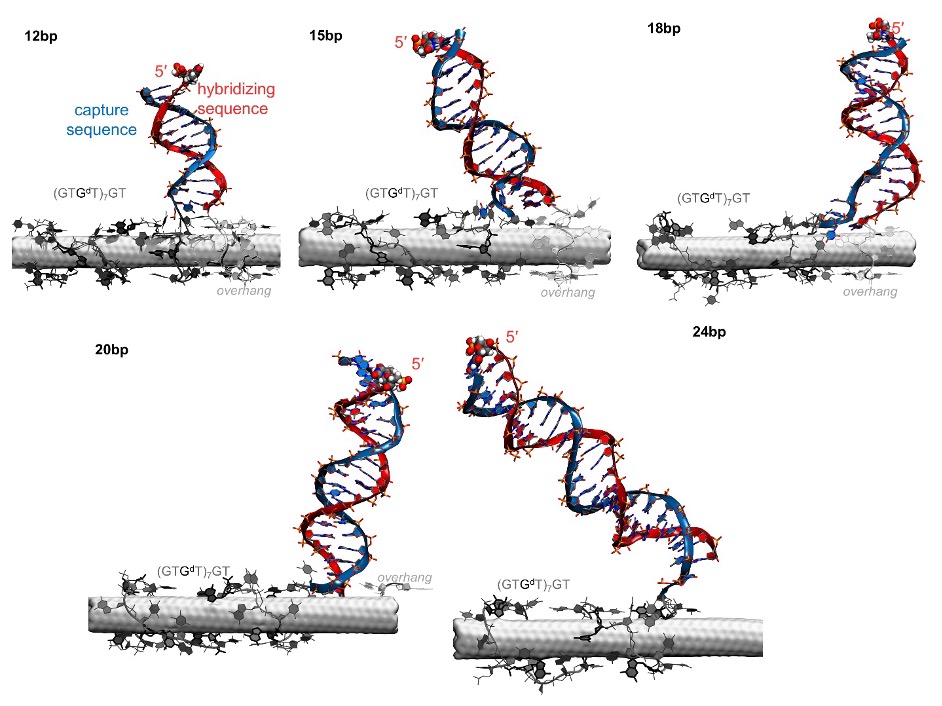


**Figure S14.** Duplex orientations at the end of molecular dynamics simulations for all the systems. Structure of the 12bp, 15bp, 18bp, 20bp and 24bp systems after ~400 ns equilibration in MD simulations. The SWCNT is shown as a white surface. The anchor sequence and the overhang segment of the complementary strand are shown in dark gray and light gray, respectively, using licorice and ribbon representations. The duplex is shown in blue (capture sequence) and red (complementary sequence) using licorice and cartoon representations. The 5′-end nucleotide of the complementary strand, which is not base-paired, is shown with van der Waals spheres. Atoms are colored as: C (gray), N (blue), O (red), P (orange), H (white).


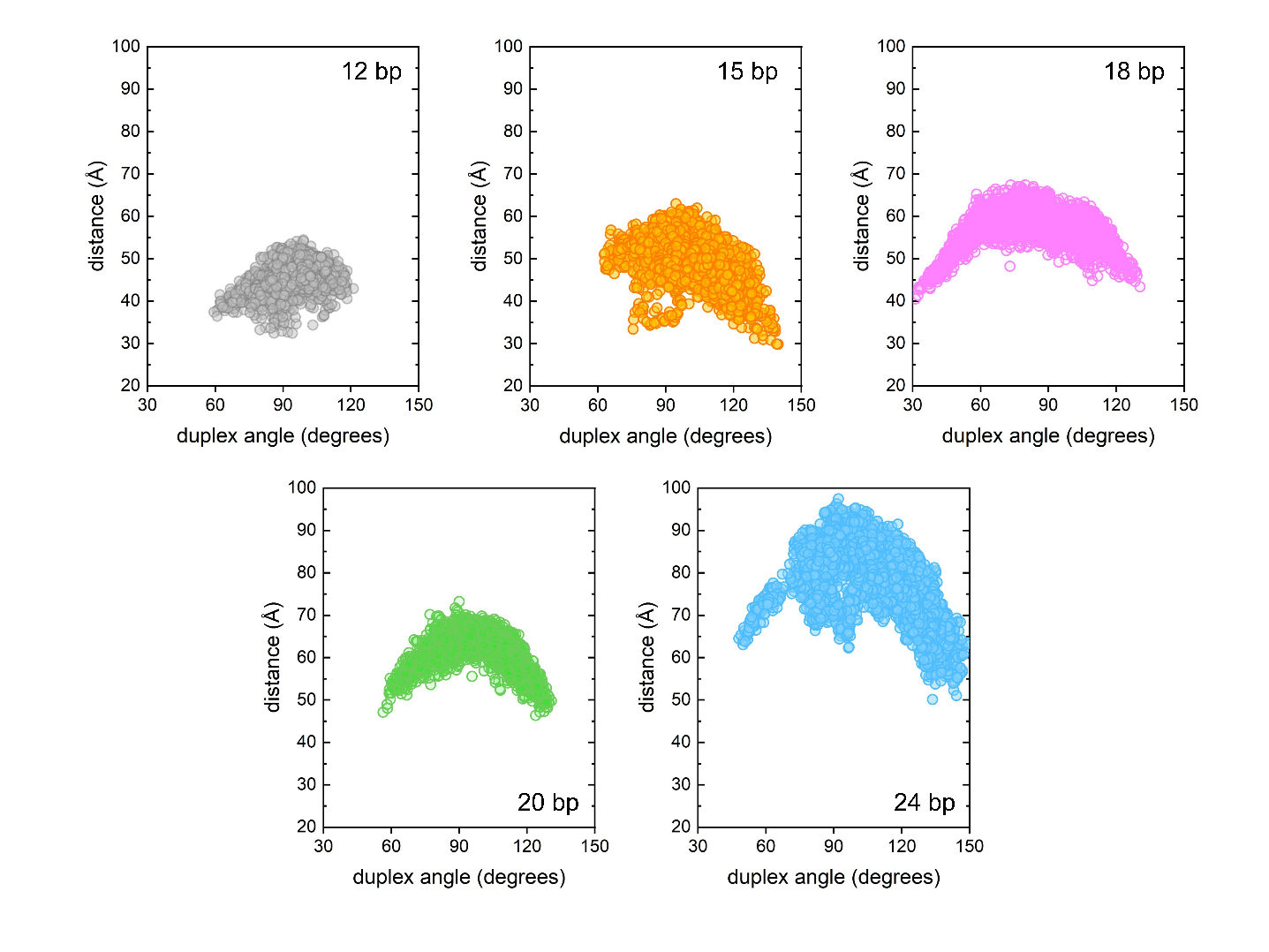


**Figure S15.** Plots of distances of duplex tips from the SWCNT surface versus the orientations of the duplexes. Distances between the 5′-end nucleotides of the hybridizing sequences and the nearest points on the SWCNT surface versus the angles between duplexes and the SWCNT axis, over 400 ns of MD simulations. The five different colors label the data from the five simulated systems.

To examine the effect of the unhybridized residual ssDNA on the dynamical and average behavior of the duplexes, we compared the behavior of DNA duplexes in systems with and without the unhybridized ssDNA segment. The results are shown in Figure 2 and Figure S16, respectively.

Across duplexes of different lengths (12–24 bp), the duplex orientation angles relative to the nanotube surface exhibited a slightly narrower distribution when the unhybridized ssDNA segment was present. This behavior likely arises from steric and electrostatic repulsion between the negatively charged duplex and the unhybridized ssDNA segment, which favors orientations closer to orthogonal relative to the nanotube surface. Although the orientation angle distributions showed shifts in the peak positions between systems, these differences were stochastic and not reproducible across independent simulations. The consistent narrowing of the distributions, rather than the exact peak positions, was therefore identified as the meaningful indicator of the unhybridized ssDNA’s effect. The narrower range reflects slightly restricted orientational flexibility of the duplex, whereas the peak shifts result from the stochastic nature of molecular–dynamics trajectories.

The distances between the duplex tip and the nanotube surface (Figures 2c and S16c) also showed comparable average values, with slightly narrower distributions in several systems containing unhybridized ssDNA. These small differences indicate that the presence of unhybridized ssDNA modestly restricted duplex motion but did not substantially alter its average position or orientation.


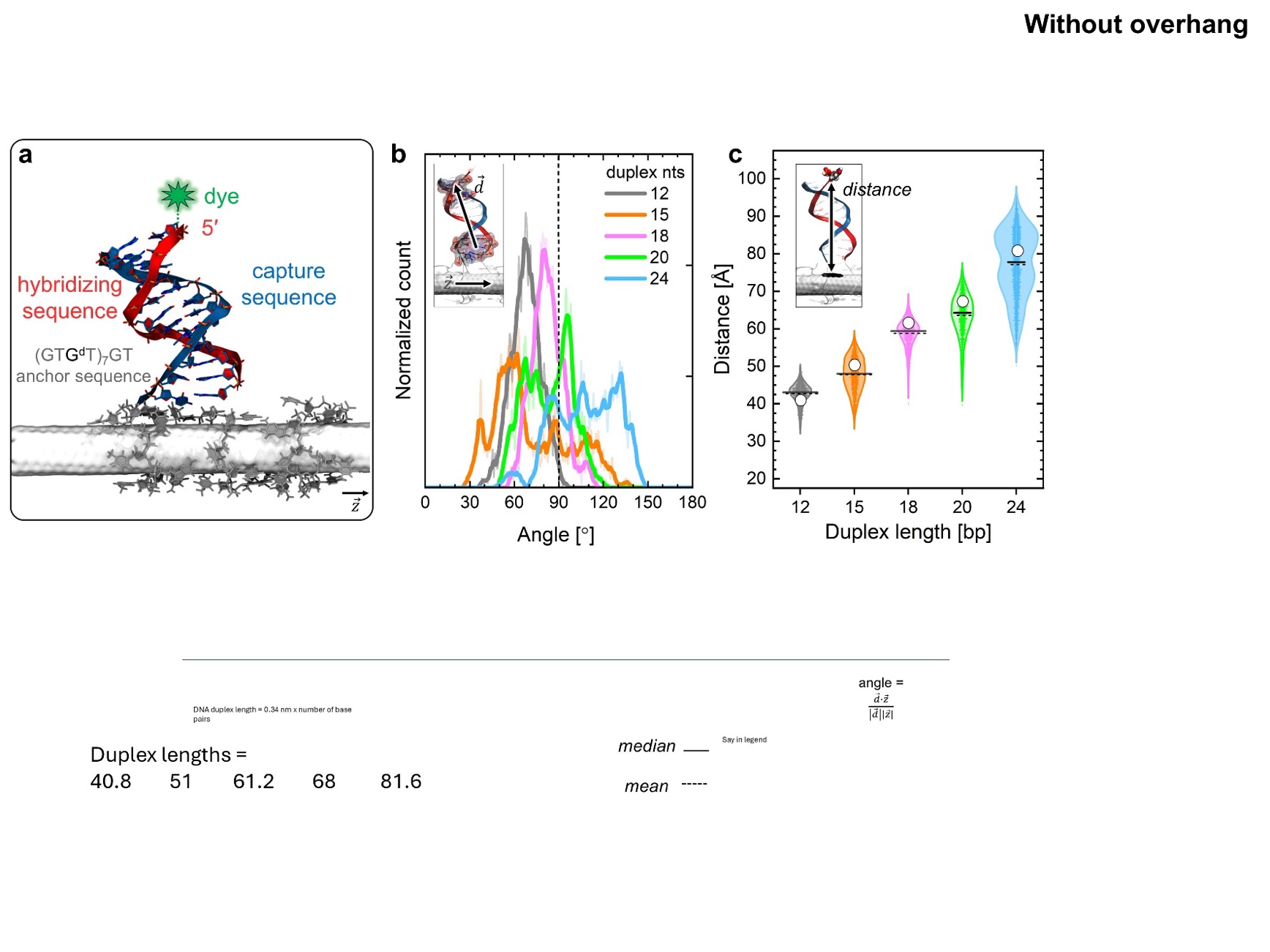


**Figure S16.** Duplex tip distances and orientations in molecular dynamics simulations for systems without unhybridized residual ssDNA. a) Structure of the S1-12bp system after 200 ns equilibration in MD simulations. The SWCNT is shown as a white surface. The anchor sequence is shown in light gray, using licorice and ribbon representations. The duplex is shown in blue (capture sequence) and red (complementary sequence) using licorice and cartoon representations. Atoms are colored as: C (gray), N (blue), O (red), P (orange), H (white). b) Angles between duplexes and the SWCNT axis over 200 ns of MD simulations. The dotted line marks the orthogonal orientation (90°). (inset) A schematic showing two vectors used to calculate angles between duplexes and the SWCNT axis using the dot product relationship. c) Distances between the 5′-end nucleotides of the complementary sequences and the nearest points on the SWCNT surface, over 200 ns of MD simulations. Empty circles mark the estimated duplex lengths, based on a 0.34 nm rise per base pair. Solid and dashed horizontal lines in violin plots indicate the median and mean values of distributions, respectively. (Inset) A schematic defining the distance measured in each system.

References:

[1] J. T. Metternich, J. A. C. Wartmann, L. Sistemich, R. Nißler, S. Herbertz, S. Kruss, *JACS* **2023**, *145*, 14776-14783.

[2] A. M. Szalai, G. Ferrari, L. Richter, J. Hartmann, M. Z. Kesici, B. Ji, K. Coshic, M. R. J. Dagleish, A. Jaeger, A. Aksimentiev, I. Tessmer, I. Kaminska, A. M. Vera, P. Tinnefeld, *Nat. Methods* **2025**, *22*, 135-144.

[3] P. Galonska, J. M. Mohr, C. A. Schrage, L. Schnitzler, S. Kruss, *J. Phys. Chem. Lett.* **2023**, *14*, 3483-3490.

[4] Y. Zheng, A. A. Alizadehmojarad, S. M. Bachilo, R. B. Weisman, *J. Phys. Chem. Lett.* **2022**, *13*, 2231–2236.

[5] Z. Lin, L. C. Beltran, Z. A. De Los Santos, Y. Li, T. Adel, J. A. Fagan, A. R. Hight Walker, E. H. Egelman, M. Zheng, *Science* **2022**, *377*, 535-539.

[6] W. Humphrey, A. Dalke, K. Schulten, *J. Mol. Graph.* **1996**, *14*, 33-38.

[7] L. L. C. Schrödinger, W. DeLano, <http://www.pymol.org/pymol>.

[8] G. Zheng, X.-J. Lu, W. K. Olson, *Nucleic Acids Res.* **2009**, *37*, W240–W246.

[9] E. E. S. Ong, J. L. Liow, *Fluid Phase Equilibr.* **2019**, *481*, 55-65.

[10] J. Huang, A. D. MacKerell, *Curr. Opin. Struct. Biol.* **2018**, *48*, 40-48.

[11] J. Huang, A. D. Mackerell, *J. Comput. Chem.* **2013**, *34*, 2135–2145.

[12] B. R. Brooks, R. E. Bruccoleri, B. D. Olafson, D. J. States, S. Swaminathan, M. Karplus, *J. Comput. Chem.* **1983**, *4*, 187-217.

[13] A. A. Alizadehmojarad, X. Zhou, A. G. Beyene, K. E. Chacon, Y. Sung, R. L. Pinals, M. P. Landry, L. Vuković, *Adv. Mater. Interfaces* **2020**, *7*, 2000353.

[14] A. G. Beyene, A. A. Alizadehmojarad, G. Dorlhiac, N. Goh, A. M. Streets, P. Král, L. Vuković, M. P. Landry, *Nano Lett.* **2018**, *18*, 6995–7003.

[15] Y. Zheng, A. A. Alizadehmojarad, S. M. Bachilo, A. B. Kolomeisky, R. B. Weisman, *ACS Nano* **2020**, *14*, 12148–12158.

[16] A. A. Alizadehmojarad, S. M. Bachilo, R. B. Weisman, *Nano Lett.* **2022**, *22*, 8203–8209.

[17] S. Chakraborty, E. Ebarguen, K. E. Chacon, M. V. Petković, L., *J. Phys. Chem. C* **2023**, *127*, 19759–19768.

[18] T. A. Darden, D. M. York, L. G. Pedersen, *J. Chem. Phys.* **1993**, *98*, 10089–10092.

[19] J. C. Phillips, D. J. Hardy, J. D. C. Maia, J. E. Stone, J. V. Ribeiro, R. C. Bernardi, R. Buch, G. Fiorin, J. Hénin, W. Jiang, R. McGreevy, M. C. R. Melo, B. K. Radak, R. D. Skeel, A. Singharoy, Y. Wang, B. Roux, A. Aksimentiev, Z. Luthey-Schulten, L. V. Kalé, K. Schulten, C. Chipot, E. Tajkhorshid, *J. Chem. Phys.* **2020**, *153*, 044130.
